# Osmolarity: A hidden factor in Nanotoxicology 

**DOI:** 10.1186/s40199-016-0146-9

**Published:** 2016-03-22

**Authors:** Saeid Moayyedi, Omid Mashinchian, Rassoul Dinarvand

**Affiliations:** Department of Medical Nanotechnology, School of Advanced Technologies in Medicine (SATiM), Tehran University of Medical Sciences, Tehran, Iran; Institute of Bioengineering, School of Life Sciences, Ecole Polytechnique Fédérale de Lausanne (EPFL), Tehran University of Medical Sciences, Lausanne, Switzerland; Nanotechnology Research Centre, Faculty of Pharmacy, Tehran University of Medical Sciences, Tehran, Iran

**Keywords:** Nanocarriers, Drug delivery, Osmotic pressure

## Abstract

In the field of drug delivery, long circulating nanocarriers in the blood have many advantages such as targeted drug delivery and sustained release. Based on our current knowledge, evaluation of the effect of long circulating nanocarriers in the blood stream on osmolarity of plasma has not been reported before. In this study, osmotic pressure developed by some commercially available nanocarriers was estimated based on Van't Hoff equation. It is noteworthy that theoretically, nanocarriers do not have any significant effect on osmolarity of plasma. However, it is worth being evaluated experimentally in order to be taken into account in future studies.

## Letters to the editor

An osmosis phenomenon is the net movement of solvent (usually water) through a semipermeable membrane from a region of high water concentration (hypoosmolar solution) to a lower water concentration (hyperosmolar solution). Adding a solute to pure water decreases water concentration in the solution. In such conditons, water molecules can diffuse from a region of low solute concentration to one with a high solute concentration [1]. The effect of diverse solutes (i.e., molecules or ions) on osmolarity is depended on the number of dissolved particles in a solution, and is not correlated to their mass. Consequently, in an equal mass ratio, macromolecules (e.g., proteins, nucleic acids, polysaccharides) have much less influence on the osmolarity of a solution in comparison with their monomeric components. For example, a gram of a polysaccharide comprised of 1,000 glucose units and a milligram of glucose have the identical effect on osmolarity. In order to prevent of an enormous increase in osmolarity inside the storage cell (e.g., hepatocyte), the fuel is stored in the form of polysaccharide (i.e., starch or glycogen) rather than glucose or other simple sugars by the cells [2].

Similarly, increasing osmolarity of plasma leads to net reabsorption of fluid from interstitial fluid into the capillaries rather than net filtration. On the other hand, increasing osmolarity of plasma leads to antidiuretic hormone secretion. Osmolarity of plasma is approximately 300 mOsm/L. Change as small as 1 % in osmolarity of plasma leads to increasing antidiuretic hormone secretion significantly. This hormone decreases the excreted volume of fluid by the kidneys. Ultimately, Due to increased blood volume, arterial pressure increases [1, 3].

In the field of drug delivery, long circulating nanocarriers in the blood have many advantages such as targeted drug delivery and sustained release [4–6]. Until now, numerous studies have been performed in the Nanotoxicology [7, 8]. Nevertheless, evaluation of the effect of long circulating nanocarriers in the blood stream on osmolarity of plasma has not been reported before.

For this reason, osmotic pressure developed by some commercially available nanocarriers per 1 L of plasma estimated based on Van't Hoff equation. Data shown in Table 1 illustrates that theoretically, nanocarriers do not have any significant effect on osmolarity of plasma. However, it is worth being evaluated experimentally in order to be taken into account in future studies.

**Table 1:** Estimation of osmotic pressure developed by some commercially available nanocarriers per 1 L of plasma

Trade name	Osmotic pressure (mm Hg)	Osmolarity (mOsm/L)
Doxil	7.0 × 10^−5^	4.0 × 10^−6^
Abraxane	2.0 × 10^−2^	1.0 × 10^−3^
AmBisome	3.2 × 10^−4^	1.7 × 10^−5^
Marqibo	3.2 × 10^−5^	1.7 × 10^−6^
DepoCyt	3.0 × 10^−5^	2.3 × 10^−6^
